# Digital self-harm – Social Media and its impact on Non-Suicidal Self-Injury and suicidal behavior. A Longitudinal Mixed Method Study

**DOI:** 10.1192/j.eurpsy.2022.868

**Published:** 2022-09-01

**Authors:** M.E. Jensen, M. Vinberg, K. Andreasson, J. Klausen, K. Joergensen, M. Nordentoft

**Affiliations:** 1 Mental Health Services in the Capital Region of Denmark, Mental Health Centre North Zealand, Hilleroed, Denmark; 2 Mental Health Services in the Capital Region of Denmark, Mental Health Center Glostrup, Glostrup, Denmark; 3 Danish Deaconess Comunity, Diakonissestiftelsen, Frederiksberg, Denmark; 4 CORE-Copenhagen Research Center for Mental Health, Mental Health Center Copenhagen, Hellerup, Denmark

**Keywords:** self-harm, social media, Mixed Method, suicidal behavior

## Abstract

**Introduction:**

Several initiatives within psychiatric nursing targets Non-suicidal self-injury (NSSI) in DK, but none targets the new phenomenon Digital Self-harm. Digital self-harm involves the use of Social media (SoMe) to harm oneself for example by communicating condescending content about and to oneself through fake profiles, seeking out conflicts to be humiliated, and consciously get others to say vicious things about themselves. Further, images are exchanged showing wounds, broken extremities, etc. and thoughts and feelings are exchanged about suicidal actions as well as methods for both self-harm and suicide actions. We do not know enough about what constitutes the problem nor do we know how to address neither the behavior nor their consequences. Due to conflicting results, more research is needed to understand how media affects NSSI as well as suicidal behavior.

**Objectives:**

The overall objective of this study is to map, at a national level, how SoMe is used as part of NSSI and suicidal behavior and get insight as to what constitutes the behavior and how we address it through three sub-studies.

**Methods:**

The study will be carried out as a mixed method study and includes a systematic review (Study 1), a qualitative part, which will be examined through interviews (Study 2), and finally a quantitative part that will be conducted through questionnaires (Study 3).

**Results:**

The project is ongoing.

**Conclusions:**

Prospects of this study are that the project will create clarity about the essence of the phenomenon of digital self-harm, how NSSI and suicidal behavior is affected and generate enough knowledge to develop interventions aiming digital self-harming and suicidal behavior.

**Disclosure:**

No significant relationships.

